# Progress in Medicine

**Published:** 1899-11-04

**Authors:** 


### Progress in Medicine.

### DISEASES OF THE RESPIRATORY TRACT.

(*Continued from page* 64.)

**Phthisis** (*continued*).—The open-air sanatorium treatment of phthisis in the British Isles is attracting much attention at the present time.[11] Philip describes the work of the Victoria Hospital for Consumption at Edinburgh. He emphasises the fact that no harm is likely to result from the exposure of patients in the open air even during the winter months. A table is given showing the number of hours spent out of doors by all patients between February 1st and April 30th, and this shows that there has not been a single day for many years on which patients were not outside for a considerable period. The results of this method of treatment have been uniformly good. There have been no untoward accidents, and an improvement in the cheerfulness and well-being of the patients. Appetite returns, constipation disappears, the power of assimilation grows, sputum lessens, night sweats and hæmophysis are rare, and the temperature and pulse rate drop. The system of "forced feeding" is not in vogue at Edinburgh, but the increase in body weight is very rapid. The Victoria Hospital is for patients of the poorer classes. The open-air treatment was adopted last year at the Throne Consumptive Hospital, near Belfast. Previous to this time, according to Calwell, the results of treatment had been very disappointing; under the new *régime* there has been a marked improvement, but this has been chiefly confined to early cases. Two patients were unable to endure the cold. The Nordrach treatment has also been adopted at the neighbouring Foster Green Hospital. Burton Fanning has been using the sanatorium treatment of phthisis for the past four years on the Norfolk coast. He advises a moderate diet only. Chronicity of disease offers greater obstacles to success than the presence of fever, for prognosis depends as much on the condition of the stomach as that of the lungs. Where amyloid changes have set in there is little hope for improvement. Sanatorium treatment is better than home treatment, but very good results can be obtained with the most unfavourable surroundings. Patients subject to bronchitis or with failing heart power should be sent to warmer climates. The Nordrach system has been given a seven years' trial at Downham by Dr. Jane Walker. Of 78 patients treated since 1892, 26 have recovered, 6 are improving, 4 going back, 9 died, 7 lost sight of, and 26 still under treatment. Nearly half the patients were of the poorer class, and could only stay for a limited period under treatment. Thurnam insists on strict adherence to the principles of Walther, and at Nordrach-on-Mendip this system is rigidly adhered to. Rest, freedom from business and friends, and pure air, plentiful and suitable food and regulated exercise are the essentials of sanatorium treatment. The patient's temperature is taken in the rectum four times daily, and the amount of exercise regulated by the pyrexia. An hour's rest before each meal is insisted upon, and every effort is made to induce the patient to take the allotted portion of food, neither fever nor dyspepsia being allowed to interfere with this. Half a litre of milk is drunk at each of the three meals. With a view to strengthening the muscular system, and especially the heart, the extent and pace of the daily walk is gradually increased until finally as much as 10 or 12 miles is covered, in the morning, without the temperature reaching 38 deg. Cent. Results have been most encouraging. Within the few months under review there have been four absolute cures, one relative cure, and all cases show improvement in weight and general health. Lucas[12] tabulates the results of the first year's work at the North London Consumption Hospital under the Nordrach *régime*. Of 183 patients treated, 43·7 per cent. showed improvement, 32·2 per cent. slight improvement, 4·3 per cent. unaltered, and 3·9 per cent. died. Hillier,[13] whose experience of open air treatment has been acquired in South Africa, states that the risks from exposure are very small, attacks of catarrh or pneumonia being practically unknown. Living in a pure atmosphere not only lessens the patient's risk of reinfection, but also intensifies and invigorates the cellular elements of the tissues, and enables them to defy the attacks of the tubercle bacillus. The patient should be warmly clothed in wool, and should rest on a long deck chair. The head should be protected, and although exposure to rain, dew, &c., may not cause injury, the wet clothes should always be changed before resting. Fog is prejudicial to most consumptives, as also are strong winds. It is necessary, therefore, to provide shelters. Actual strong draughts should be avoided, and in the winter months the patient's room may need artificial heating, but the temperature should not be raised above 50 deg. to 55 deg. Fahr. Sea voyages are of doubtful benefit. If taken, a deck cabin is essential. High altitudes and dry climates are preferable for many cases, but the one essential is pure air.

Weber[14] gives the following directions for the climatic treatment of phthisis: Weakly subjects do best in warm, sunny, sheltered places such as the Riviera; strong, torpid constitutions benefit by mountain or sea air. High altitudes and sea voyages are suitable for cases in which the disease is limited to one apex, and in which there is little fever. If in such cases there be fever home treatment is best. With extensive disease and slight fever a warm seaside locality should be chosen. Long journeys are contraindicated in advanced febrile cases. Chronic, quiescent, and cases with albuminuria without fever do well at the Riviera or in Egypt. If phthisis be complicated by diabetes high altitudes must be avoided. Pau or Bournemouth are suited for catarrhal cases; Madeira, the Canaries, and Arcachon for emphysematous cases. Amongst mountain climates in order of merit are placed the Peruvian Andes (Jauja and Quito), Rocky Mountains, Colorado, Swiss Alps, and South African highlands. The Riviera and Egypt, especially Helouan, have low elevation, and suitable warm climate for consumptives. Madeira, the Canaries, the Eastern Riviera, and Sicily are valuable seaside resorts. The voyages to Australia, New Zealand, and the Cape of Good Hope are best for consumptives. The subject of rest and exercise for consumptives is dealt with by Ransom.[15] In the earlier days of the disease rest is imperative, and rest in the recumbent posture has the following advantages: The body weight is increased, the blood improves in quality and oxygenation, the circulation in the extremities is facilitated, the temperature falls, and repose is ensured to the ribs over the injured lung. For these reasons rest in the recumbent position should be enforced, especially after meals, and no exercise which will quicken the muscles of forced respiration allowed. Dyspeptic patients and those losing weight should be kept at rest. Gentle walking exercise for ten minutes every two hours is sufficient in the early stages of convalescence; later may come short rides on a bicycle, moderate games of croquet, or "putting" on a green, carriage exercise, &c. There must be no loss of breath and no over-fatigue. Hill climbing is not to be encouraged, even when convalescence is well established, and no form of sport likely to lead to excitement or rise of blood-pressure. Reading, drawing, music, and light manual tasks should fill the bulk of the day. Fowler[16] found obsolete tubercle of lung in nine per cent. of 1,943 necropsies, either as pigmented tubercles which have undergone fibrosis, as caseous masses in fibrous capsules, or as quiescent cavities. Arrest is brought about by increasing the resisting power of the individual and the Nordrach treatment is the best known means of obtaining this end. He points out that the larger the amount of food administered the less is the significance to be attached to a gain in weight as evidence of the arrest of the disease. The temperature should be taken in the rectum, for the oral temperature in the evening is 1 to 2 deg. Fahr. lower than the rectal, whilst in the morning the rectal is slightly the lower.

**Medicinal Treatment.**—At the Berlin Congress many persons spoke of the value of various forms of medicinal treatment. Kobet recommended pyramidon for the reduction of temperature. Coghill had obtained good results with guiacol combined with strychnine, and Landerer with subcutaneous injections of cinnamic acid. Cervello advocated the inhalation of formaldehyde vapour. Petruscky spoke favourably of tuberculin treatment; but Brieger stated that no antituberculous serum has hitherto been proved to be in any sense specific. Ambler [7] reports 106 cases of phthisis treated by Fisch's antiphthisic serum. There was apparent cure in 39 per cent. of cases, and improvement in 29 per cent. About ·5 c. centimetre of serum was used at each injection, and 77 c. centimetres was the average amount given in all. Williams and Horrocks obtained serum from a horse injected with tuberculin, and this appeared to be useful in early cases of phthisis with limited lesion. Freudenthal's[18] results with such a serum, however, are not encouraging. M'Call Anderson[19] records a series of cases treated by tuberculin with good results. He states, however, that as the bacilli alone are attacked and not the predisposing cause there is a great tendency to relapse, and the treatment should be combined with pure air, good food, codliver oil, tonics, &c. This is the experience of Stubbert,[20] who used the serum prepared by the United States Bio-Chemical Laboratory. Of 90 cases, 24 per cent. were cured and 36 per cent. improved. The same writer speaks highly of ichthyol in doses of 30 grains three times daily. Amongst other methods of medication we may mention the use of formalin, as recommended by Lardner Green.[21] One drachm of formalin, four drachms of glycerine, and four ounces of distilled water used as a spray four times in 24 hours, the inhalations lasting from 10 to 15 minutes each. Macgregor has obtained good results even in advanced cases of phthisis from the administration of chinosol, five grains three times daily after food. Subcutaneous injections of camphorated oil or of camphor (about three-tenths of a grain) are recommended by Alexander. The treatment must be kept up for from four to six weeks.

[11] Practitioner, July, 1899. [12] Report of North Lond. Hosp. for Consump., 1899. [13] Practitioner, Aug., 1899. [14] Brit. Med. Jour., June 3, 1899. [15] Brit. Med. Jour., July 22, 1899. [16] Lancet, Aug. 12, 1899. [17] Med. Rec., June 17, 1899. [18] Canad. Jour., April, 1899. [19] Glasgow Med. Jour., May, 1899. [20] Med. Rec., May 20, 1899. [21] Lancet, Aug. 19, 1899.

### DISEASES OF THE DIGESTIVE ORGANS.

**Œsophagus.**—In a paper on the anatomy of this organ D. T. Coffey[1] mentions that the glands in the human œsophagus are distributed in vertical rows of about 13 each, the majority occurring in the upper half of the tube, while the unstriped muscular fibres surround it nearly to the top. Zahorsky[2] records a case of a diverticulum which was situated just above the diaphragm instead of opposite the cricoid, which is the usual spot. The patient had difficulty in swallowing, and, after several mouthfuls, by an extraordinary effort appeared to get the food into his stomach, but at times regurgitated quantities of food without hydrochloric acid or peptones. After swallowing a capsule of potassium iodide none could be detected in the saliva. A rigid bougie sometimes passed into the stomach, but a soft tube generally stopped at 15¾ in., and, if a meal had been taken, about 14 ounces could be withdrawn by it. An area of dulness was often observed in the left infrascapular region. Operation was deemed impracticable, and the patient remained for some years in fair health.

**Septic Dyspepsia.**—The serious effect of bad teeth on the digestion through the absorption of toxins, pus, and micro-organisms is emphasised by Fitzgerald,[3] who points out that various stomach disorders may thus be set up, especially chronic gastritis, as well as some forms of septicæmia, from the growth of micro-organisms in neglected mouths. These results are particularly to be dreaded in pyorrhœa alveolaris, which is produced in cases of chronic catarrh of the mouth from nasal obstruction, in syphilis, iodism, gout, and other diseases.

**Anorexia.**—In cases of loss of appetite of an obstinate type from various causes orexine has been recommended for some years. The tannate is an improvement, because it is tasteless, and may be given in four to eight grain doses twice daily with marked results in anæmia, early phthisis, neurasthenia, the vomiting of pregnancy, and in many diseases of children. It is counterindicated in malignant and amyloid degeneration of the stomach, acute fevers, and similar conditions. A collection of papers on the subject is given in "Merck's Archives," May, 1899, where T. A. Goldmann is quoted as claiming for it not only a most remarkable power of increasing the appetite, but also that of increasing the secretion of hydrochloric acid and the motor power of the stomach, so that digestion is much quickened and the eructations and sense of fulness after a meal are lost. Numerous instances of its successful use are recorded, and the abstractor of these papers has seen a remarkable instance where a woman of 80 years, who had long subsisted on scanty liquid food, commenced to take solid nourishment with a most vigorous appetite upon using orexine.

**Ulcer of the Stomach.**—The assertion of Würternitz that rectal feeding was useless in this disease has been disproved by Ziasko.[4] It was stated that the reflex effect of enemata was to increase the secretion of gastric juice, which would be disadvantageous to an ulcerated stomach. Chemical investigation in 10 patients, however, showed that enemata actually diminish the acid secreted to a considerable degree. It would, however, appear, from the investigations of Krokiewitz,[5] that instances of ulcer occur in which free acid is entirely absent, which is confirmed by Einhorn. He thinks this is especially the case when the ulcers are multiple. The question of diagnosis from cancer would then be difficult, but he relies upon counting the blood cells, which are always diminished in malignant cachexia, and are normal in most cases of ulcer. A case of perforation with subsequent recovery without operation is reported by C. J. Whitby.[6] The stomach was fortunately empty at the time, and rectal feeding was being carried out as hæmatemesis had occurred. Suddenly severe localised pain and tenderness came on with other symptoms of perforation of definite character. Operation was deferred, and the condition of the patient gradually improved, but a severe hæmorrhage during his recovery was almost fatal. More than two quarts of saline solution were injected into the veins, and at last the patient rallied, regaining his ordinary strength and weight.

Achilles Rose[7] directs attention to the gastralgia which often continues for a long period after the healing of an ulcer, whether from cicatricial pressure on a nerve or from adhesions, and recommends the introduction of $CO_2$ into the stomach by a tube as a useful remedy. In the discussion of his paper Einhorn spoke in favour of the galvanic current, and thought that the symptom could not be referred to the existence of a movable kidney as has been suggested. In the treatment of ulcer he preferred milk, or milk diluted with barley water, to peptonised milk, from its greater power of absorbing acid when this is present in excess. The hyperchlorydria is often only a result and not a cause of ulcer.

[1] Brit. Med. Jour., July 22. [2] New York Med. Jour., July 8. [3] Clin. Jour., Mar. 8. [4] La Semaine Med., May 11. [5] Internat. Med. Mag., Aug. [6] Lancet, June 24. [7] Post Graduate, July.

